# Comparative Effect of MSC Secretome to MSC Co-culture on Cardiomyocyte Gene Expression Under Hypoxic Conditions *in vitro*

**DOI:** 10.3389/fbioe.2020.502213

**Published:** 2020-10-05

**Authors:** Nina Kastner, Julia Mester-Tonczar, Johannes Winkler, Denise Traxler, Andreas Spannbauer, Beate M. Rüger, Georg Goliasch, Noemi Pavo, Mariann Gyöngyösi, Katrin Zlabinger

**Affiliations:** ^1^Department of Cardiology, Medical University of Vienna, Vienna, Austria; ^2^Department of Blood Group Serology and Transfusion Medicine, Medical University of Vienna, Vienna, Austria

**Keywords:** human cardiomyocytes, hypoxia, regeneration, cell therapy, MSC secretome

## Abstract

**Introduction:**

Despite major leaps in regenerative medicine, the regeneration of cardiomyocytes after ischemic conditions remains to elucidate. It is crucial to understand hypoxia induced cellular mechanisms to provide advanced treatment options, including the use of stem cell paracrine factors for myocardial regeneration.

**Materials and Methods:**

In this study, the regenerative potential of hypoxic human cardiomyocytes (group Hyp-CMC) *in vitro* was evaluated when co-cultured with human bone-marrow derived MSC (group Hyp-CMC-MSC) or stimulated with the secretome of MSC (group Hyp-CMC-S_MSC_). The secretome of normoxic MSC and CMC, and the hypoxic CMC was analyzed with a cytokine panel. Gene expression changes of *HIF-1*α, proliferation marker *Ki-67* and cytokinesis marker *RhoA* over different reoxygenation time periods of 4, 8, 24, 48, and 72 h were analyzed in comparison to normoxic CMC and MSC. Further, the proinflammatory cytokine IL-18 protein expression change, metabolic activity and proliferation was assessed in all experimental setups.

**Results and Conclusion:**

*HIF-1*α was persistently overexpressed in Hyp-CMC-S_MSC_ as compared to Hyp-CMC (except at 72 h). Hyp-CMC-MSC showed a weaker *HIF-1*α expression than Hyp-CMC-S_MSC_ in most tested time points, except after 8 h. The *Ki-67* expression showed the strongest upregulation in Hyp-CMC after 24 and 48 h incubation, then returned to baseline level, while a temporary increase in *Ki-67* expression in Hyp-CMC-MSC at 4 and 8 h and at 48 h in Hyp-CMC-S_MSC_ could be observed. *RhoA* was increased in normoxic MSCs and in Hyp-CMC-S_MSC_ over time, but not in Hyp-CMC-MSC. A temporary increase in IL-18 protein expression was detected in Hyp-CMC-SMSC and Hyp-CMC. Our study demonstrates timely dynamic changes in expression of different ischemia and regeneration-related genes of CMCs, depending from the culture condition, with stronger expression of *HIF-1*α, *RhoA* and IL-18 if the hypoxic CMC were subjected to the secretome of MSCs.

## Introduction

Myocardial ischemia leads to permanent cell damage after 20 to 60 min (Silbernagl and Lang, 2000), depending on the *in vitro* and *in vivo* conditions. Cardiovascular diseases account for approximately a third of all deaths (Benjamin et al., 2017) globally and though long-term survival has improved over the last decades, regenerative treatment options for lost myocytes still remain limited (Puymirat et al., 2017). In 2001 the first approaches of human regenerative therapy have been tested with myoblasts, leading to further cell transplantation trials (Menasché et al., 2001). Since then several different regeneration possibilities have been explored in heart failure treatment, such as re-activation of endogenous cardiomyocyte proliferation via cell reprograming, activation of vascularization with growth factors and immunomodulation (Cahill et al., 2017). The initial research focus was on replacement of cardiomyocytes (CMC) by stem cell delivery, however, since the hypothesis of stem cells differentiating into cardiomyocytes was refuted, the focus shifted to stimulation of endogenous cardiac repair (Orlic et al., 2001; Murry et al., 2004). Extracellular vesicles and paracrine factors released by stem cells may influence and improve cardiac repair, since they carry proliferative and growth factors (Cahill et al., 2017).

Mesenchymal stem cells (MSC) are adult stem cells that are multipotent and can differentiate into adipocytes, osteoblasts and chondrocytes (Beresford et al., 1992; Pittenger et al., 1999). Transdifferentiation of MSCs to CMCs is still heavily debated (Szaraz et al., 2017; Guo et al., 2018). The first discovery of MSC was in bone marrow (Prockop, 1997), however, they have been found in most post-natal tissues (da Silva Meirelles et al., 2006). Bone marrow (Kern et al., 2006; Pendleton et al., 2013), adipose tissue (Zuk et al., 2002; Kern et al., 2006), the umbilical cord (Sarugaser et al., 2005; Beeravolu et al., 2017) and the placenta (Pelekanos et al., 2016) are the most commonly used sources for MSC isolation. The International Society of Cellular Therapy has defined certain criteria for characterization of isolated MSC, consisting of ability to differentiate into adipocytes, osteoblast and chondrocytes *in vitro*, plastic adhesion and expression of CD105 +, CD90 +, CD73 +, CD45-, CD34-, CD14- or CD11b-, CD79α - or CD19- and HLA-DR- (Dominici et al., 2006).

The first use of bone marrow derived cells in clinical trials for heart regeneration was by Assmus et al. (2002) by injecting a variety of cell types residing in bone morrow. Selected bone marrow-derived MSC were first studied in cardiac research in mice by Yoon et al. in 2005 (Yoon et al., 2005). The majority of first-generation clinical trials, however, focused on injection of unselected bone-marrow origin mononuclear cells or hematopoietic stem cells, rather than MSC (Gyöngyösi et al., 2015). In 2014 one of the first clinical trial with bone marrow derived MSC was completed by Karantalis et al. (2014; Karantalis et al., 2014). The general understanding was that stem cells can modulate cell behavior and proliferation of the cardiomyocytes, however, the mechanism was not fully understood, which lead to the hypothesis of stem cell differentiation to CMC (Yoon et al., 2005). Recently, the hypothesis shifted to the reparative effect of paracrine factors that contain proliferation-inducing factors, including transcription, growth, angiogenic and immunosuppressive factors (Thum et al., 2005; Safari et al., 2016; Shafei et al., 2017). Paracrine factors are theorized to induce re-entry into the cell cycle of CMCs, however, the regulation mechanism has not been fully understood (Shafei et al., 2017). The benefit of using just excreted secretome and not cells themselves is the lowered risk of an immune reaction when using allogenic MSC. It is considered to be safe to use MSC due to the low expression of MHC molecules, however, in recent years several studies have encountered anti-donor immune responses non-etheless (Lohan et al., 2017).

In contrast to the initial assumption, that intracoronary infusion of bone-marrow cells induces myocardial regeneration in reperfused acute myocardial infarction, patient individual data-based meta-analysis has revealed no significant benefit of this treatment, and the research has focused on other cell types and interventions (Gyöngyösi et al., 2015).

In this study we aimed to simulate the reperfused acute myocardial infarction *in vitro* and evaluated the effect of MSCs or their secretome on the temporary hypoxic CMC. We have supposed that just using secretome of MSC (instead of cells) has a similar or better benefit for CMC survival and proliferation. Accordingly, we have evaluated the expression of the angiogenic marker *HIF-1*α, the cytokinesis marker *RhoA* and the proliferation marker *Ki-67*, and the proliferation/metabolic activity (EZ4U kit, MTT assay, BrdU assay) of the CMCs and proinflammatory cytokine IL-18 protein levels.

## Materials and Methods

### Study Design

**FIGURE 1:**
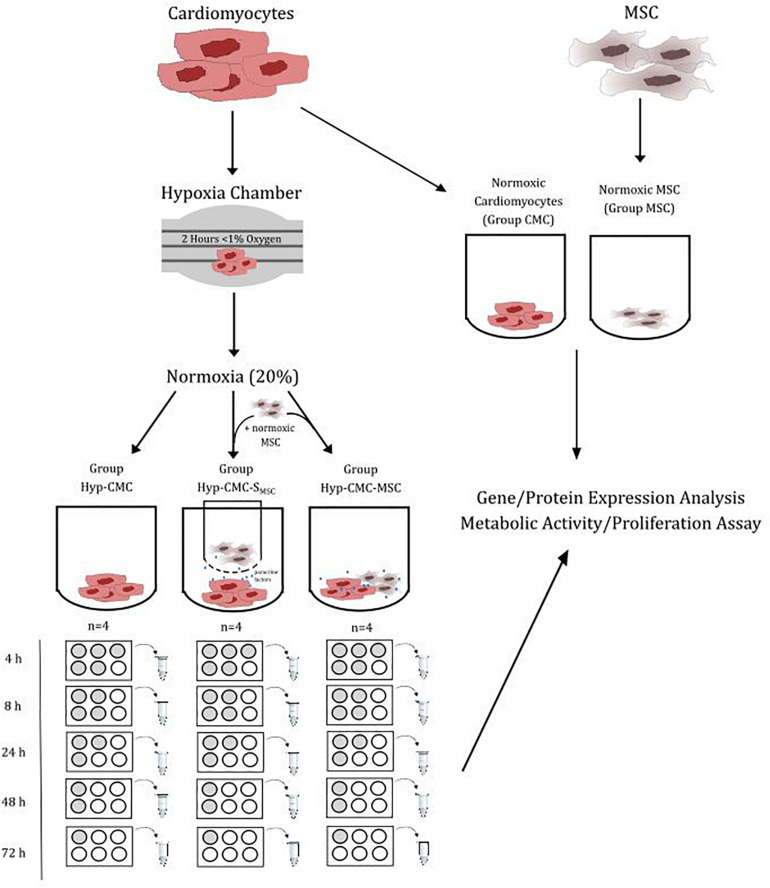
Overview of the study design setup. CMC were exposed to < 1% oxygen level for 2 h. After restoration of the normoxic condition, the CMCs were separated in three groups: Unstimulated hypoxic CMC (Hyp-CMC), CMC stimulated with MSC secretome (Hyp-CMC-S_MSC_) and CMC stimulated directly with MSC (Hyp-CMC-MSC). The samples of each setup were analyzed after the respective reoxygenation period. Additionally, normoxic CMC (CMC) and normoxic MSC (MSC) were analyzed. Each setup was evaluated using gene/protein analysis and a proliferation assay. MSC – Mesenchymal stem cells. CMC – cardiomyocytes. Hyp – hypoxic.

The protein expression and proliferation of hypoxic human CMC (Hyp-CMCs) was tested in co-culture with human MSCs directly (Hyp-CMC-MSC) or in culture with secretome of human MSCs (Hyp-CMC-S_MSC_) via 3 μm transwells (Greiner Bio-One International GmbH, Austria). Additionally, unstimulated hypoxic CMCs were evaluated. CMCs (PromoCell GmbH, Germany) were cultured in 6-well plates (TPP, Switzerland) and used for experiments at 95% confluency. Human MSCs (EK1193/2015) were kindly provided from the Department of Blood Group Serology and Transfusion Medicine, Medical University of Vienna (Rüger et al., 2018). The setup of the study is summarized in Figure 1.

CMCs were cultured in Basal Medium (PromoCell GmbH, Germany) supplemented with Growth Medium SupplementMix, (PromoCell GmbH, Germany) with final concentrations of FBS 0.05 mL/mL, Epidermal Growth Factor (recombinant) 0.5 ng/mL, Basic Fibroblast Growth Factor (recombinant human) 2 ng/mL and insulin (recombinant human) 5 μg/mL. 5⋅10^4^ CMC were seeded into each well of 6-well plate and medium was changed 24 h before to M199 (Sigma-Aldrich, Germany) supplied with 1% P/S (Sigma-Aldrich, Germany). M199 + 1% P/S was changed again right before conduction of the experiment.

MSCs were cultured in Dulbecco’s Modified Eagle Medium DMEM HG 4.5 g/L glucose (Sigma-Aldrich, Germany) supplemented with 10% Fetal Bovine Serum FBS (Biochrom GmbH, Germany) and 1% Penicillin/Streptomycin. Medium was exchanged to M199 + 1% P/S 24 h before MSCs were used in experiments.

CMCs were exposed to a gas mixture of nitrogen and oxygen via a hypoxia chamber (Billups-Rothenberg Inc., United States) for 2 h and an oxygen level of under 1%. M199 + 1% P/S was again changed right after, inducing normoxic conditions (simulating myocardial reperfusion). Afterward Hyp-CMC were classified to one of the three experimental groups, unstimulated CMC (Hyp-CMC), directly stimulated with MSC (Hyp-CMC-MSC) and indirectly stimulated with MSC secretome (Hyp-CMC-S_MSC_). Unstimulated CMC were lysed after the respective time period without further processing. Directly stimulated CMCs were co-cultured with MSC by adding 5⋅10^4^ MSC into the well right after hypoxia and then also lysed after the respective follow-up time period. Indirectly stimulated CMC were co-cultured with MSC by transferring a transwell with 5⋅10^4^ pre-seeded MSC to the well right after hypoxia and then also lysed after the respective time period. Additionally, 5⋅10^4^ unstimulated MSC were seeded into two wells of a 6-well-plate and lysed after 24 h culturing in serum free M199 medium. Tested time periods after reoxygenation were 4, 8, 24, 48, and 72 h. Additionally, samples were evaluated directly after hypoxia and before hypoxia as control. Each setup was carried out with *n* = 4.

### RNA Isolation and cDNA Synthesis

Cells were lysed with 350 μL RLT Buffer (QIAGEN GmbH, Germany) per well with 1% β-mercaptoethanol (Sigma-Aldrich, Germany). RNA was automatically isolated from cell lysates with the QIACube (QIAGEN GmbH, Germany) using QIAgen miRNeasy Kit (QIAGEN GmbH, Germany). Isolated RNA was measured with a NanoDrop^TM^ 3300 Fluorospectrometer (Thermo Fisher Scientific, United States) and diluted, respectively, with RNAse free water (QIAGEN GmbH, Germany) to a final concentration of 41.67 ng/μL. Further 2 μL gDNA Wipeout Buffer (QIAGEN GmbH, Germany) was added to 12 μL sample, equaling 500 ng RNA, and incubated at 42°C for two minutes. Reverse Transcriptase MM, QuantiScript RT Buffer and RT Primer Mix from the QuantiTect Reverse Transcription Kit (QIAGEN GmbH, Germany) were then added to the samples, which were then incubated at 42°C for 15 min for cDNA synthesis. The Reverse Transcriptase was then inactivated via incubation at 95°C for 3 min.

### Gene Expression Analysis via qPCR

cDNA samples were diluted to a concentration of 2.5 ng/μL with RNAse free water. The PCR plate was stored on a cooling block and 4 μL sample equaling 10 ng RNA, 10 μL QuantiTect SYBR Green PCR MM (QIAGEN GmbH, Germany), 2 μL Primer (10 μM) forward, 2 μL Primer (10 μM) reverse and 2 μL RNAse free water was added. Following primer were designed with PrimerBlast from NCBI and obtained from Microsynth Austria GmbH, Austria: *HIF-1*α fwd ACC TGA GCC TAA CAG TCC CAG TG, *HIF-1*α rev TTC TTT GCC TCT GTG TCT TCA GCA A (Tm = 63°C, 104 bp); *Ki-67* fwd CCA CAC TGT GTC GTC GTT TG, *Ki-67* rev CCG TGC GCT TAT CCA TTC A (Tm = 59°C, 123 bp); *RhoA* fwd CCC AAT GTG CCC ATC ATC CT, *RhoA* rev TGG TTT TAC TGG CTC CTG CT (Tm = 60°C, 102 bp); *HPRT* fwd CCC AGC GTC GTG ATT AGT GA, *HPRT* rev ATC TCG AGC AAG CCG TTC AG (Tm = 60°C, 141 bp). The PCR plate was briefly centrifuged at 300 × *g* when all components were added. The PCR was carried out in the QuantStudio 5 PCR cycler (Applied Biosystems by Thermo Fisher Scientific, United States) and the according software Analysis Software v1.4.1. The setup consisted of a 15-min hold stage at 95°C at the beginning, followed by 40 cycles of 94°C for 15 s (Denaturation), 60°C for 30–60 s (Annealing) and 72°C for 30 s (Elongation). Data was collected in the Elongation Step. After the PCR stage, a melt curve was obtained. Gene expression was analyzed using the 2^(–ΔΔ*Ct*)^ method.

### Characterization of CMCs and MSCs via Immunofluorescence Staining

Normoxic MSCs and normoxic CMCs were characterized with an immunofluorescence staining of specific markers.

CMCs were indirectly labeled with α-Sarcomeric-Actinin, BNP, Connexin 43 and Troponin-T, heavy chain cardiac Myosin, Nkx2.5 (Abcam plc., United Kingdom). CMCs were characterized for each experiment setup (CMC, Hyp-CMC, Hyp-CMC-SMSC and Hyp-CMC-MSCS). CMSs were seeded into 96-well plates (TPP, Switzerland) and 48-well plates (TPP, Switzerland). Experiments as described in 2.1 and subsequent staining was performed when 80% confluency was reached. CMCs were fixed in 4% paraformaldehyde (Merck, Germany) for 15 min. After removing the fixative solution, the CMCs were washed two times with PBS. Primary antibody solutions were prepared by appropriately diluting the respective antibodies in permeabilization buffer, consisting of 3% non-fat dry milk (Sigma Aldrich, St. Louis, MO, United States) with 0.1% Triton X-100 (Sigma Aldrich, St. Louis, MO, United States) in PBS. CMCs were incubated with the respective antibody solution for 2 h and were then washed twice with PBS. Then the secondary antibody solution with Goat-anti-Rabbit 488, Goat-anti-Mouse FITC, Donkey-anti-Goat 488 (Abcam plc., United Kingdom), in permeabilization buffer was added to the cells for 2 h. A 1:2500 Hoechst in PBS working solution was added for 20 min without discarding the secondary antibody solution. CMCs were then counterstained for 10 min with a 1:40 Phalloidin (Thermo Fisher Scientific, United States) working solution in PBS after washing with PBS twice. CMCs were again washed two times with PBS and then and stored at 4°C in 100 μL PBS per well.

MSCs were seeded into 8-well-slips, pre-coated with 0.1% porcine gelatin (Sigma-Aldrich, Germany). Staining was performed when 80% confluency was reached. MSCs were therefore fixed with 2.5% formalin (Merck, Germany) for 15 min and permeabilized with methanol (Fisher Chemical, United Kingdom) for three minutes. 0.1% Tween-20 (Sigma-Aldrich, Germany) in PBS was added as blocking buffer for 15 min. MSC were directly labeled with CD105, CD90, CD44, and CD29 (EXBIO Praha a.s., Czechia) with the, respectively, diluted antibody for an hour. A 1:5000 Hoechst (Sigma-Aldrich, Germany) in PBS working solution was added for five minutes. The slip was mounted with fluoroshield mounting medium (Abcam, United Kingdom) and stored at 4°C.

Images were taken on the Olympus IX83 Inverted Microscope (OLYMPUS EUROPA SE & CO. KG, Germany) using cellSens imaging software.

### EZ4U Proliferation Assay

Evaluation of proliferation was additionally performed with an EZ4U Proliferation Assay (Biomedica Medizinprodukte GmbH, Austria), which is based on the reduction of tetrazolium salt to colored formazan that can then be measured and is directly proportional to the mitochondrial oxidative capacity of the living cells. The substrate was prepared according to manufacturer protocol and was then added to the respective wells 4 h before readout of the time-point. The readout was performed with the Tecan Sunrise plate reader (Tecan Group, Switzerland) at the wavelength of 450 nm, 620 nm as reference wavelength.

### MTT Assay

Evaluation of metabolic activity was performed with an MTT Assay (Thermo Fisher Scientific, United States) according to the manufacturer protocol using DSMO. The readout was performed with the Tecan Sunrise plate reader (Tecan Group, Switzerland) at the wavelength of 540 nm as indicated by the manufacturer.

### BrdU Assay

Cell cycle progression was measured with a BrdU Assay (Exalpha Biologicals Inc., United States), which relies on [3H] thymidine incorporation as cells enter S phase. The protocol was performed according to the manufacturer protocol. The readout was performed with the Tecan Sunrise plate reader (Tecan Group, Switzerland) at the wavelength of 450 nm and 550 as reference as indicated by the manufacturer.

### Cytokine Evaluation of CMC and MSC Secretome

A cytokine analysis was performed with the Proteome Profiler Human Cytokine Array Kit (R&D systems, United States). Samples of cell secretome (2 mL serum-free M199 medium) were taken from a 6-well plate with approximately 5⋅10^4^ cells of the respective cell type (MSC, CMC, Hyp-CMC). 1 mL of the samples was used for the analysis. The assay was performed according to the manufacturer protocol. Readout was performed by adding 1 mL Novex^TM^ ECL Chemiluminescent Substrate Reagent Kit (Thermo Fisher Scientific, United States) 1:1 according to manufacturer protocol and imagining in the membranes on the ChemiSmart-3600 (Peqlab Biotechnologie GmbH, Germany) after 3 min of incubation. The images were then analyzed with the fiji software to evaluate the intensity of the signal.

### IL-18 ELISA

An IL-18 ELISA BMS672 (affymetrix eBioscience, United States) was performed with cell lysates to determine protein expression. Cells were lysed for 10 min with trypsin after the respective regeneration time and stored at −80°C. The ELISA was then performed according to the manufacturer protocol. The standard curve was fitted with a 4-parameter logarithmic curve fit.

### Statistical Analysis

Statistical analysis was conducted in Prism 5 for Windows v5.01 (GraphPad Software, Inc.) using one-way ANOVA (Kruskal-Wallis test) and groups were compared with the Dunn’s Multiple Comparison Test.

## Results

### Characterization of MSC Showed Characteristic Stem Cell Markers and CMC Showed Characteristic Cardiac Lineage Associated Markers

**FIGURE 2:**
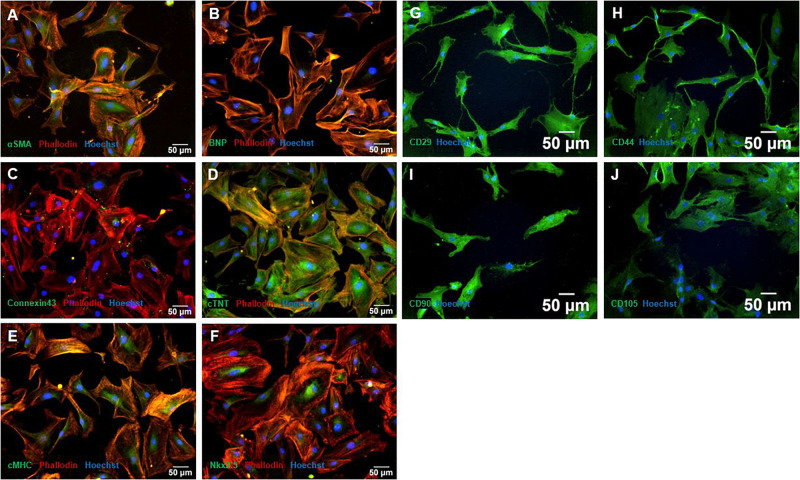
Characterization with immunofluorescence staining of MSC and CMC. CMC showed typical cardiac lineage marker expression of αSMA **(A)**, BNP **(B)**, Connexin 43 **(C)**, cTNT **(D)**, cMHC **(E)**, and Nkx2.5 **(F)**. MSC showed the expression of characteristic stem cell markers CD29 **(G)**, CD44 **(H)**, CD90 **(I)**, and CD105 **(J)**. Cells were counterstained with Hoechst. MSC – Mesenchymal stem cells. CMC – cardiomyocytes. cTNT – Cardiac Troponin T. cMHC - Cardiac Heavy Chain Myosin. αSMA - α-sarcomeric-Actinin.

As seen in Figures 2A–F, CMCs showed positive expression of all cardiac lineage associated markers BNP (Figure 2B), Connexin 43 (Figure 2C), cTNT (Figure 2D), cMHC (Figure 2E), and Nkx2.5 (Figure 2D), indicating a CMC phenotype. Further, αSMA was weakly expressed. Connexin 43 was mainly expressed in gap junctions, cTNT as shown in Figure 2D showed structural features of CMCs. BNP and cMHC was expressed consistently throughout the cells (Figures 2A,E).

In hypoxia treated CMCs, the expression of αSMA and BNP could not be detected or only weakly in all experimental setups, all other marker showed expression throughout all groups and timepoints (Supplementary Figures 1–5).

Immunofluorescence staining of naïve MSCs showed positive expression of specific stem cell markers CD29, CD44, CD90 and CD105 (Figures 2G–J). CD105 occasionally showed weaker expression, CD29, CD44, and CD90 were expressed throughout all cells (Figure 2J).

### Cytokine Analysis of Secretome of Unstimulated Normoxic CMC and MSC and Hyp-CMC Revealed Specific Cytokine Release

**FIGURE 3:**
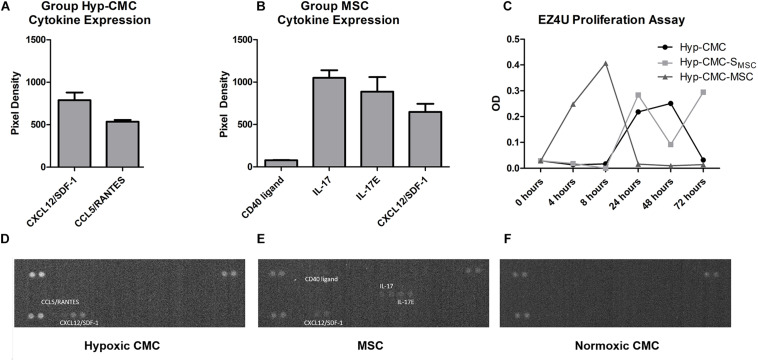
Cytokine expression and EZ4U proliferation assay readout in unstimulated hypoxic and normoxic CMC and normoxic MSC. (**A** and **D**) Cytokine expression of Hyp-CMC secretome, showing detectable readout of CXCL12/SDF-1 and CCL5/RANTES. (**B** and **E**) Cytokine expression of normoxic MSC secretome, with detectable readout of CD40 ligand, IL-17, IL-17E, and CXCL12/SDF-1. **(C)** EZ4U proliferation assay of the control CMC and MSC and of the samples taken from each of the three experiment groups unstimulated hypoxic CMC (group Hyp-CMC), CMC stimulated with MSC secretome (group Hyp-CMC-S_MSC_) and CMC stimulated directly with MSC (group Hyp-C-MSC) after the respective tested time period of 4, 8, 24, 48, and 72 h. **(F)** No detectable cytokine expression in normoxic CMC. MSC – Mesenchymal stem cells. CMC – cardiomyocytes. Hyp – hypoxic.

The cytokine array was carried out to determine secretome content and to characterize important key factors. Secrectome analysis for cytokines revealed an expression of CCL5/RANTES (534 ± 15.0) and CXCL12/SDF-1 (3682 ± 238.5) in secretome of Hyp-CMC (Figures 3A,D). In secretome of normoxic CMC (group CMC), there was no detectable cytokine expression (Figure 3F). Secretome of normoxic MSC (group MSC) showed cytokine expression for CD40L (78 ± 2.0), IL-17 (1050 ± 63.0), IL-17E (888.5 ± 120.5), and CXCL12/SDF-1 (648.5 ± 67.5) (Figures 3B,E). Evaluation of all experimental setups and time points was out of the scope of this study.

### EZ4U Assay Showed Elevated CMC Metabolic Activity in Long-Term Regeneration When Stimulated With MSC Secretome

The EZ4U assay (Figure 3C) showed an increased metabolic activity in Hyp-CMC-MSC after 4 h (0.249) and 8 h (0.407), followed by rapid decrease with OD values of 0.016 (24 h), 0.010 (48 h), and 0.014 (72 h). Metabolic activity of Hyp-CMC and Hyp-CMC-S_MSC_ increased first 24 h after reoxygenation and remained undulant high in Hyp-CMC-S_MSC_ at 48 and 72 h (OD values: 0.284 – 0.092 – 0.295, respectively), while it decreased in Hyp-CMC (OD values: 0.218 – 0.251 – 0.032, respectively).

### MTT and BrdU Assay Showed no Significant Differences in Proliferation and Metabolic Activity Between Groups

**FIGURE 4:**
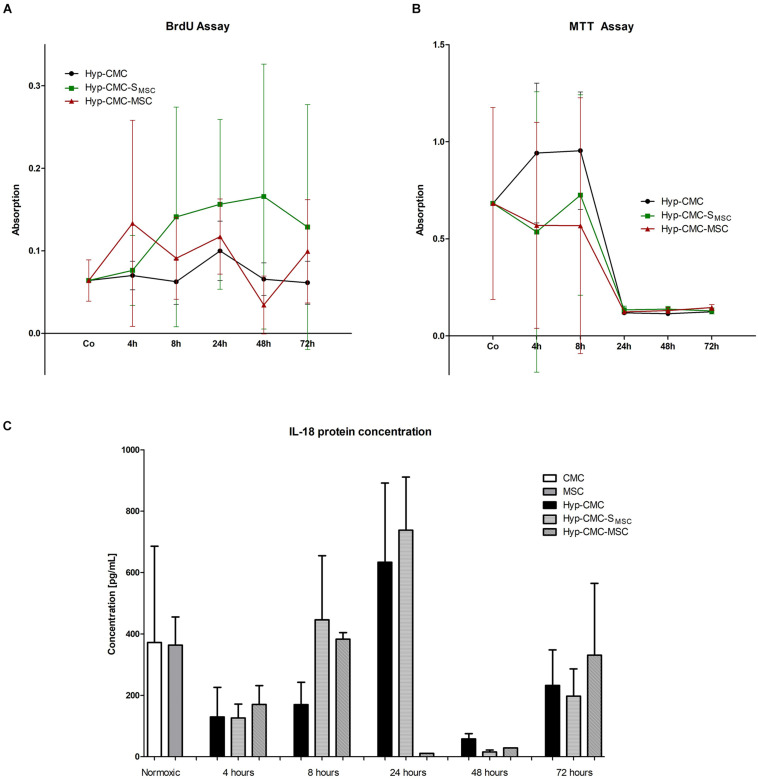
**(A–C)** BrdU and MTT assay, IL-18 protein concentration analysis with ELISA of the three experimental groups: unstimulated hypoxic CMC (group Hyp-CMC), CMC stimulated with MSC secretome (group Hyp-CMC-S_MSC_) and CMC stimulated directly with MSC (group Hyp-CMC-MSC) after the respective reoxygenation period of 4, 8, 24, 48, and 72 h. IL-18 protein concentration of CMC and MSC were also evaluated as control. Each stimulation method and respective time point was analyzed with one-way ANOVA (Kruskal-Wallis test) and groups were compared with the Dunn’s Multiple Comparison Test. *p < 0.05, **p < 0.01, *n* = 2 technical replicates (Il-18), *n* = 6 technical replicates (BrdU and MTT). MSC – Mesenchymal stem cells. CMC – cardiomyocytes. Hyp – hypoxic.

As shown in Figure 4B, metabolic activity asses with MTT varied between time points and throughout the experimental groups of Hyp-CMC, Hyp-CMC-MSC and Hyp-CMC-S_*MSC*,_ with a drop after 24 h in all groups. The group comparison did not reveal significant differences between the groups. The proliferation asses with a BrdU assay (Figure 4A) also showed no significant difference between the groups, however, a trend toward higher proliferation in Hyp-CMC-S_MSC_ was observed.

### HIF-1α, Ki-67, and RhoA Expression Showed Elevated CMC Proliferation When Stimulated With MSC Secretome

**FIGURE 5:**
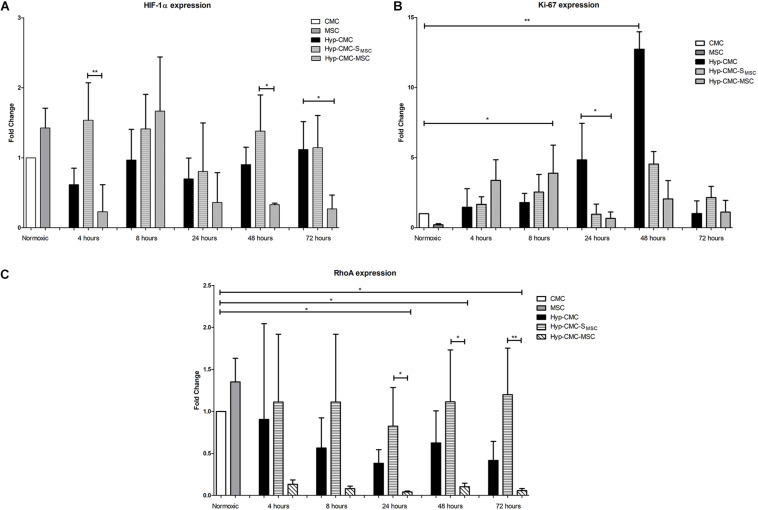
Gene expression analysis of **(A)** *HIF-1*α, **(B)** *Ki-67*, and **(C)** *Rho-A* with RT-qPCR of the control CMC and MSC and of the samples taken from each of the three experimental groups: unstimulated hypoxic CMC (group Hyp-CMC), CMC stimulated with MSC secretome (group Hyp-CMC-S_MSC_) and CMC stimulated directly with MSC (group Hyp-CMC-MSC) after the respective reoxygenation period of 4, 8, 24, 48, and 72 h. Values are relative to normoxic gene expression. *HPRT* was used as reference gene. The expression of each stimulation method was compared to the control group of the respective time point with one-way ANOVA (Kruskal-Wallis test) and groups were compared with the Dunn’s Multiple Comparison Test. *p < 0.05, **p < 0.01, *n* = 4 technical replicates. MSC – Mesenchymal stem cells. CMC – cardiomyocytes. Hyp – hypoxic.

*HIF-1*α gene expression (Figure 5A), *Ki-67* gene expression (Figure 5B) and *RhoA* gene expression (Figure 5C) showed significant differences among the experimental groups.

*HIF-1*α gene expression showed a trend toward downregulation in directly stimulated CMCs (Hyp-CMC-MSC) in comparison to Hyp-CMC-S_MSC_, reaching significance at 72 h. When comparing Hyp-CMC-MSC with Hyp-CMC-S_MSC_, the secretome treatment showed a significantly (*p* < 0.05) higher *HIF-1*α expression at 4, 48, and 72 h. Hyp-CMC-S_MSC_ showed a trend toward upregulation of *HIF-1*α expression as compared with the Hyp-CMC in all follow-ups.

Hyp-CMC showed a significant upregulation of *Ki-67* expression at 24 h when compared to the other treatment groups and remained high at 48 h before decreasing after 72 h. Similar trend toward increase in expression of *Ki-67* in both stimulated groups (Hyp-CMC-SMSC and Hyp-CMC-S_MSC_) were observed with less extent at 8 and 48 h, with decrease to almost baseline levels at 72 h.

*RhoA* gene expression showed a slight upregulation in Hyp-CMC-S_MSC_ after 4 and 8 h and a trend toward downregulation in Hyp-CMC-MSC. Hyp-CMC-S_MSC_ *RhoA* expression then showed a significant upregulation when compared to Hyp-CMC-MSC. Hyp-CMC-MSC showed a significant downregulation in Hyp-CMC-MSC from 24–72 h when compared to normoxic CMC. Hyp-CMC showed a slight downregulation at all timepoints.

### IL-18 Protein Concentration Showed Different Inflammatory Cytokine Signaling Throughout Reoxygenation Time Points and Type of Stimulation

As demonstrated in Figure 4C, Il-18 protein concentration varied between time points and throughout the experimental groups of Hyp-CMC, Hyp-CMC-MSC, and Hyp-CMC-S_*MSC*,_ with an increase at 8 and 24 h with a drop after 48 h. The group comparison did not reveal significant differences between the groups.

## Discussion

Here we show, that MSC secretome might provide a better treatment option after hypoxic events when compared to direct MSC cell therapy, based on our *in vitro* analysis. *In vitro* co-culture of human CMCs subjected to ischemia followed by normoxia (simulating ischemia/reperfusion) with human MSCs did not reveal substantial changes in *HIF-1*α, *Ki-67* and *RhoA* expression, which is line with the clinical observation that even a direct contact of ischemic injured CMC with reparative MSCs does not induce robust angiogenic or proliferative processes of the CMCs (Gyöngyösi et al., 2015). Subjecting the ischemia-affected CMC to secretome, time-dependent increase of *HIF-1*α and *RhoA* expression could be detected, indicating that cell-free therapy might be considered as a more viable option for cardiac regeneration (Pavo et al., 2014; Roura et al., 2017). In contrast to our study simulating acute myocardial ischemia and reperfusion, recent studies demonstrated that MSC show cardioprotective effects and can trigger cell-cycle progression in murine cardiomyocytes in diverse other conditions, such as chronic ischemia, or cardiomyopathies (Ezquer et al., 2015; Huang et al., 2019).

*HIF-1*α is considered as a hypoxic marker, that is linked with cell survival when and after enduring hypoxic conditions. It serves as a modulator of various pathways that allow the cell to switch metabolism, accommodate to pH variations and activate cell protective mechanisms (Weidemann and Johnson, 2008). When *HIF-1*α is upregulated, the cell has a better chance of avoiding apoptosis or necrosis, which is favorable for cardiac repair (Semenza, 2014). As *HIF-1*α showed to be generally more strongly expressed in CMCs stimulated with MSC secretome (Hyp-CMC-S_MSC_) than in CMCs stimulated with MSCs directly (Hyp-CMC-MSC), the assumption of better cell survival hints benefits of a treatment with solely excreted secretome.

Expression of *Ki-67* is linked to proliferation, since *Ki-67* is upregulated during mitosis and the G_2_ phase (Sobecki et al., 2017). Interestingly, the strongest upregulation of *Ki-67* expression was measured in unstimulated Hyp-CMCs, indicating an impairment of proliferation by the stimulation at 24 and 48 h. However, *Ki-67* has been described to vary in expression throughout cell types, presenting a more graded proliferation marker, rather than a binary proliferation marker (Miller et al., 2018). Additionally, *Ki-67* was also discovered to be upregulated in cardiomyocyte endoreduplication, without a correlation with *de novo* cardiomyogenesis (Zebrowski and Engel, 2013; Drenckhahn et al., 2015; Alvarez et al., 2019). Therefore, the expression of a cytokinesis marker (*RhoA*) and a proliferation and metabolic assay (EZ4U) was additionally performed to validate *Ki-67* expression due to proliferation.

The EZ4U assay showed a similar relation of proliferation like *Ki-67* in Hyp-CMC, as proliferation peaked after 24 and 48 h. Additionally, in Hyp-CMC-MSC, proliferation peaked after 4 and 8 h, showing similarities to *Ki-67* expression. In Hyp-CMC-S_MSC_ *Ki-67* expression and proliferation evaluated with the EZ4U assay showed a similar course, however, the peak of *Ki-67* expression after 48 h showed a time delay in the EZ4U assay after 72 h. The BrdU assay and the MTT assay showed no significant changes when CMCs were stimulated in comparison to Hyp-CMC, however, a trend to higher proliferating cells in group hyp-CMC-S_MSC_ was observed. *RhoA* expression showed a generally low expression in Hyp-CMC-MSC and a generally high expression in Hyp-CMC-S_MSC_. Further, Hyp-CMC showed a moderate *RhoA* expression throughout all reoxygenation periods. Since *RhoA* is highly expressed during anaphase, telophase and especially cytokinesis (Chircop, 2014), the readout of expression changes can be attributed to cell division and proliferation, rather than CMC endoreduplication. When comparing *RhoA* and *Ki-67* expression, some discrepancies can be observed, especially in unstimulated CMC (Hyp-CMC), which show a high *Ki-67* expression after 24 and 48 h. This may be attributed due to higher endoreduplication, as it has been described that CMC without any stimulation do not significantly replicate after ischemia (Van Amerongen and Engel, 2008). When reviewing expression changes of *HIF-1*α, *Ki-67*, and *RhoA* and the proliferation assay, the general pro-survival and proliferation inducing effect of MSC secretome can be concluded.

The secretome analysis further revealed pro-inflammatory cytokine release (CD40L, IL-17, IL-17E, and CXCL12/SDF-1) in MSC secretome, which were completely missing in normoxic CMC secretome. CD40 ligand are expressed on non-inflammatory cells in inflammatory states and induce a wide variety of immunological signaling, like T-cell induction (Elgueta et al., 2009). CXCL12/SDF-1 has been described as a crucial factor for stem cell recruitment after hypoxic cardiac injury, but adverse effects on remodeling processes have also been associated with its upregulated release (Mühlstedt et al., 2016). Similarly, the effect of IL-17 and its variant IL-17E in myocardial infarction has revealed contradictory data on outcome; however, a general understanding that IL-17 induces CMC apoptosis leading to iNOS and free radical release subsequently leading to leukocyte accumulation and cardiac repair at the target site (Carreau et al., 2011; Pietrowski et al., 2011; Mora-Ruíz et al., 2019). Hyp-CMC secretome also revealed cytokine expression of CCL5/RANTES and CXCL12/SDF-1. CCL5/RANTES signaling recruits leukocytes to the site and generally act pro-inflammatory, however, there has been some contradicting data, whether higher concentrations of CCL5/RANTES show a positive or negative effect after acute myocardial infarction (Badacz et al., 2019). Additionally, IL-18 protein concentration, linked with the regulation of cardiomyocyte hypertrophy and extracellular matrix remodeling (O’Brien et al., 2014), was generally slightly higher or equally high in Hyp-CMC-S_MSC_ in relation to the control.

Study limitations. The study was carried out *in vitro* on CMCs that showed a not fully mature characteristic. The lack of beating in serum-free culture medium indicates that (even though stated by the manufacturer) the cells were not fully mature (Boheler et al., 2002). Immature CMCs show a higher self-regeneration possibility than adult CMCs, which may influence the results (Hesse et al., 2018). Additionally, CMCs in culture only tolerated 2 h of hypoxia, before becoming severely apoptotic, making accurate analysis unattainable. Therefore, a short hypoxia time was chosen for the experiments. Further in the group stimulated with MSCs directly (Hyp-CMC-MSC), the separation of CMCs and MSCs for gene expression analysis could not be carried out. However, the gene expression in MSC were analyzed separately. The cells showed a *HIF-1*α upregulation and *Ki-67* downregulation when compared to the same normoxic control (CMC), which concludes that there is indeed a different gene regulation in MSC which influences the measurements in directly co-cultured samples.

A further deep analysis of the molecular processes was out of scope of this work. For example, investigations on oxidative stress due to ischemic damage and antioxidative enzymes (Kurian et al., 2016; González-Montero et al., 2018; Peoples et al., 2019), as well as ATP consumption (Casey and Arthur, 2000; Wu et al., 2008; McDougal and Dewey, 2017) have already been investigated and published several times; we did not intend to repeat literature data. We did not measure cardiac function recovery parameters in *in vitro* cell culture, albeit cardiac markers, such as troponin T could indicate a cardiac injury, and restoration of ischemic cardiac injury. We further did not include western blot analysis for measuring protein expression, as protein concentrations were too low to obtain a reliable result.

In conclusion the treatment with MSC secretome showed to be a comparable and even better treatment option in most parameter setups and show potential for usage as regenerative therapy after myocardial ischemia.

## Data Availability Statement

The datasets generated for this study are available on request to the corresponding author.

## Ethics Statement

Human bone marrow MSC (EK1193/2015) were used to perform this study, isolated by BR, MSc. All donors provided written informed consent.

## Author Contributions

NK, KZ, JW, JM-T, DT, and MG: planning of the study. NK: conducting of the experiments. NK, JM-T, AS, and KZ: analysis. MG: funding acquisition. MG, BR, and NP: resources. NK and KZ: writing. NP, GG, MG, KZ, and NK: review and editing. All authors reviewed and contributed to the article and approved the submitted version.

## Conflict of Interest

The authors declare that the research was conducted in the absence of any commercial or financial relationships that could be construed as a potential conflict of interest.

## Abbreviations

α SMA: α-sarcomeric -Actinin
MSC: Mesenchymal stem cells
cMHC: Cardiac Heavy Chain Myosin
CMC: Cardiomyocytes
cTNT: Cardiac Troponin T
HIF-1 α: Hypoxia-inducible factor 1 α
HPRT: Hypoxanthin guanine phosphoribosyltransferase
Hyp: Hypoxic
P/S: Penicillin/Streptomycin
RT-qPCR: Reverse transcriptase quantitative polymerase chain reaction.
