# Supplementary material for: Comparative Effect of MSC Secretome to MSC Co-culture on Cardiomyocyte Gene Expression Under Hypoxic Conditions in vitro
Source: Front Bioeng Biotechnol. 2020 Oct 5;8:502213. doi: 10.3389/fbioe.2020.502213 (PMC7571272; doi:10.3389/fbioe.2020.502213)
Supplement: Supplementary file 1 [file Data_Sheet_1.docx]

Supplementary Material

# Supplementary Figures

A complete characterization of CMCs after each experimental setup including unstimulated hypoxic CMC (group Hyp-CMC), CMC stimulated with MSC secretome (group Hyp-CMC-S_MSC_) and CMC stimulated directly with MSC (group Hyp-CMC-MSC) was carried out. Figures for each timepoint (4h, 8h, 24h, 48h and 72h) are provided in Supplementary Figure 1-5.


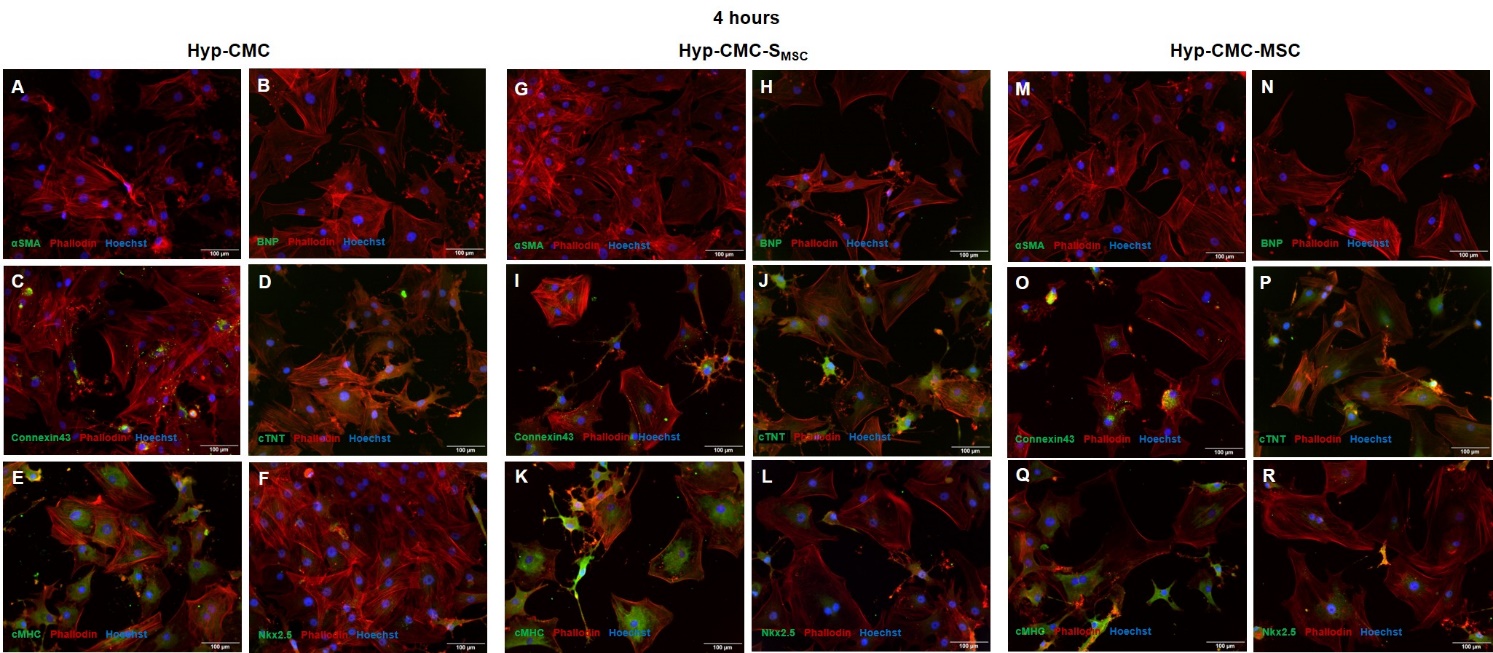


**Supplementary Figure 1:** **Characterization with immunofluorescence staining of CMC after 4 hours reoxygenation** from each of the three experimental groups: unstimulated hypoxic CMC (group Hyp-CMC, A-F), CMC stimulated with MSC secretome (group Hyp-CMC-S_MSC,_ G-L) and CMC stimulated directly with MSC (group Hyp-CMC-MSC, M-R). CMC showed no expression of αSMA (A, G, M) and BNP (B, H, N). Linage specific markers Connexin 43 (C, I, O), cTNT (D, J, P), cMHC (E, K, Q) and Nkx2.5 (F, L, R) were expressed throughout all setups. Cells were counterstained with Hoechst. MSC – Mesenchymal stem cells. CMC – cardiomyocytes. cTNT – Cardiac Troponin T. cMHC - Cardiac Heavy Chain Myosin. αSMA - α-sarcomeric-Actinin.


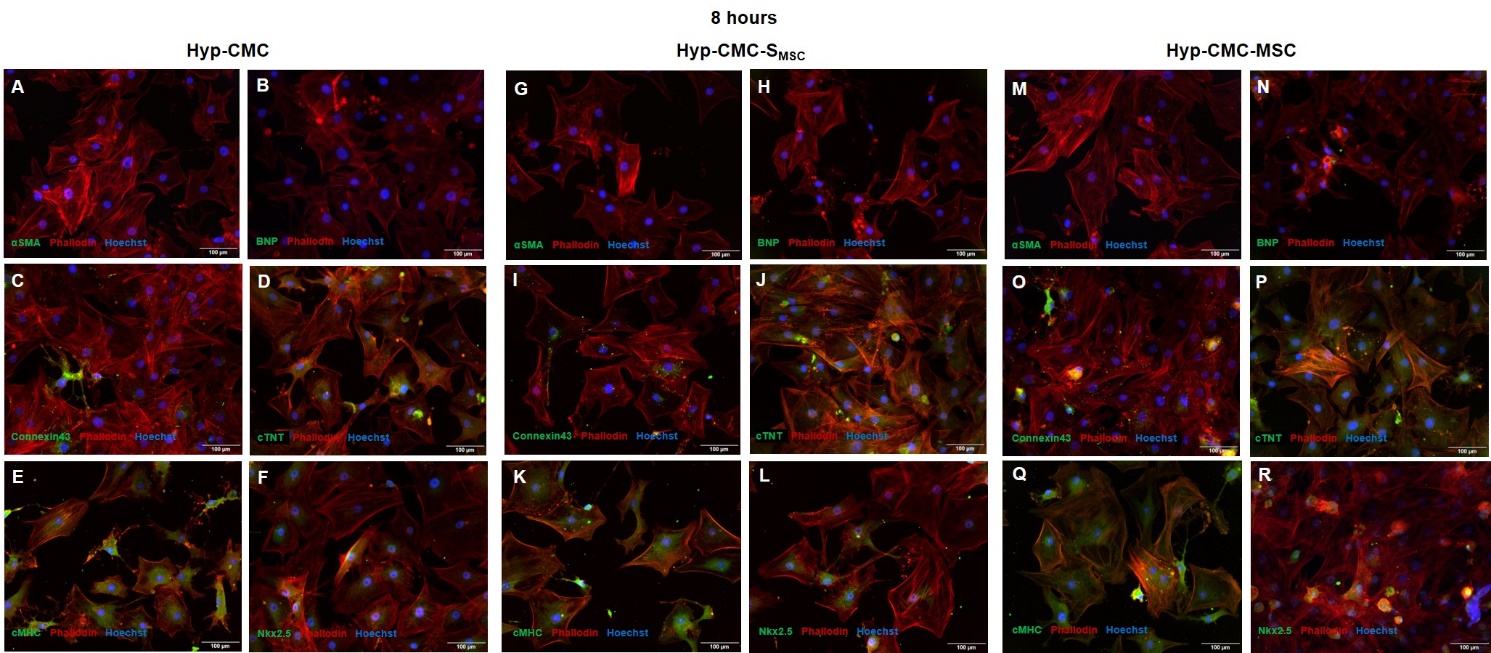


**Supplementary Figure 2:** **Characterization with immunofluorescence staining of CMC after 8 hours reoxygenation** from each of the three experimental groups: unstimulated hypoxic CMC (group Hyp-CMC, A-F), CMC stimulated with MSC secretome (group Hyp-CMC-S_MSC,_ G-L) and CMC stimulated directly with MSC (group Hyp-CMC-MSC, M-R). CMC showed no expression of αSMA (A, G, M) and BNP (B, H, N). Linage specific markers Connexin 43 (C, I, O), cTNT (D, J, P), cMHC (E, K, Q) and Nkx2.5 (F, L, R) were expressed throughout all setups. Cells were counterstained with Hoechst. MSC – Mesenchymal stem cells. CMC – cardiomyocytes. cTNT – Cardiac Troponin T. cMHC - Cardiac Heavy Chain Myosin. αSMA - α-sarcomeric-Actinin.


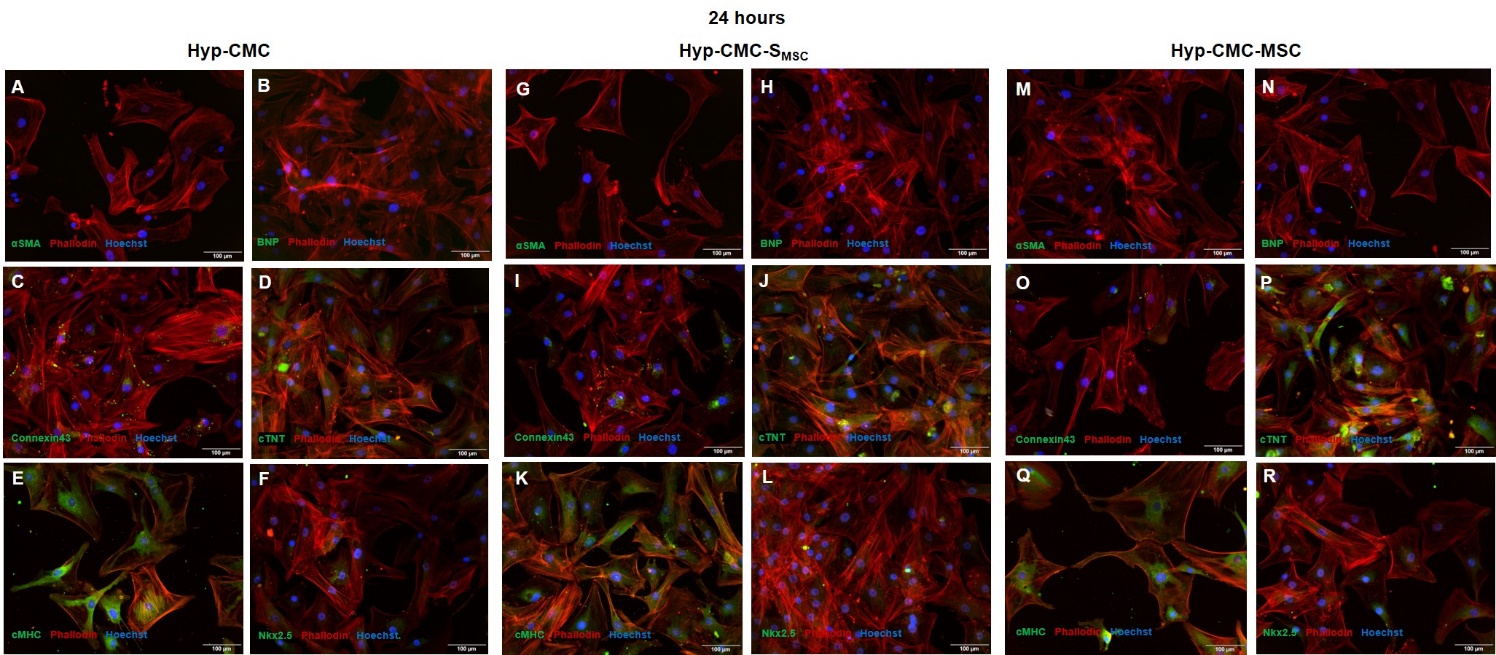


**Supplementary Figure 3:** **Characterization with immunofluorescence staining of CMC after 4 hours reoxygenation** from each of the three experimental groups: unstimulated hypoxic CMC (group Hyp-CMC, A-F), CMC stimulated with MSC secretome (group Hyp-CMC-S_MSC,_ G-L) and CMC stimulated directly with MSC (group Hyp-CMC-MSC, M-R). CMC showed no expression of αSMA (A, G, M) and BNP (B, H, N). Linage specific markers Connexin 43 (C, I, O), cTNT (D, J, P), cMHC (E, K, Q) and Nkx2.5 (F, L, R) were expressed throughout all setups. Cells were counterstained with Hoechst. MSC – Mesenchymal stem cells. CMC – cardiomyocytes. cTNT – Cardiac Troponin T. cMHC - Cardiac Heavy Chain Myosin. αSMA - α-sarcomeric-Actinin.


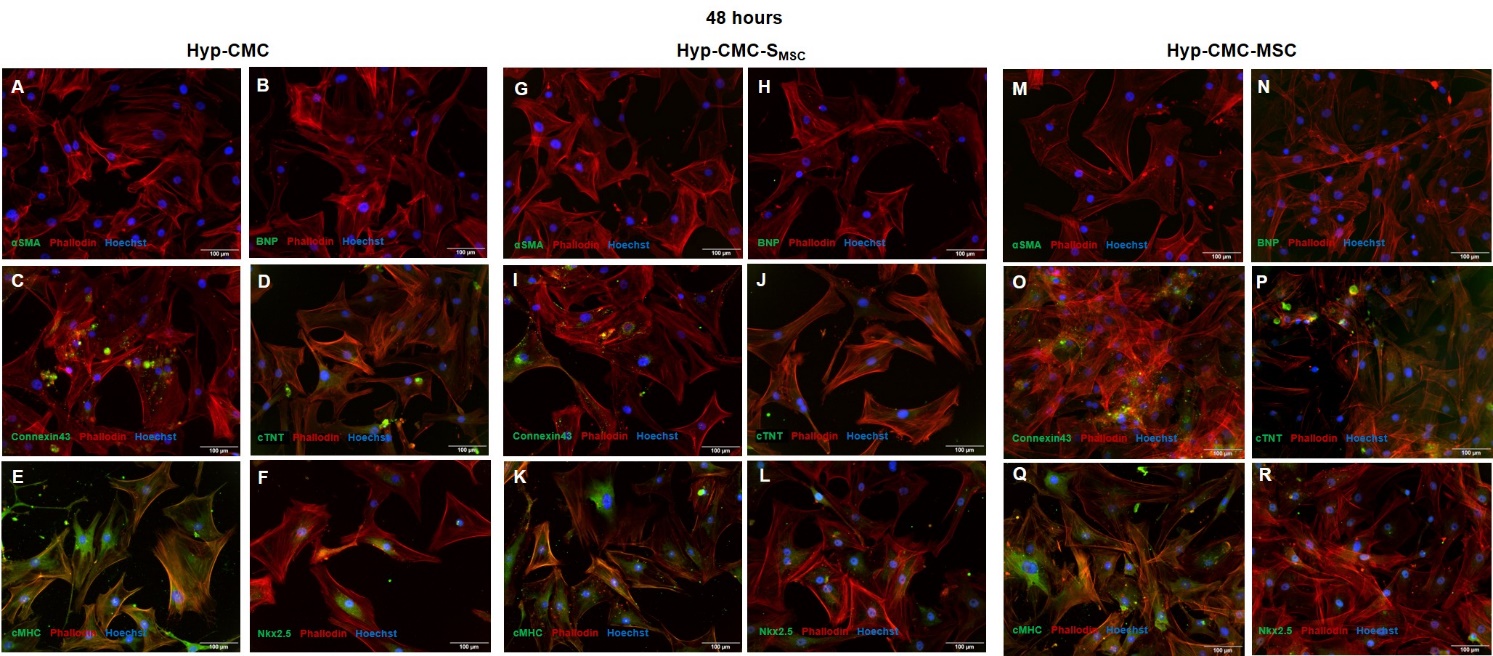


**Supplementary Figure 4:** **Characterization with immunofluorescence staining of CMC after 48 hours reoxygenation** from each of the three experimental groups: unstimulated hypoxic CMC (group Hyp-CMC, A-F), CMC stimulated with MSC secretome (group Hyp-CMC-S_MSC,_ G-L) and CMC stimulated directly with MSC (group Hyp-CMC-MSC, M-R). CMC showed no expression of αSMA (A, G, M) and BNP (B, H, N). Linage specific markers Connexin 43 (C, I, O), cTNT (D, J, P), cMHC (E, K, Q) and Nkx2.5 (F, L, R) were expressed throughout all setups. Cells were counterstained with Hoechst. MSC – Mesenchymal stem cells. CMC – cardiomyocytes. cTNT – Cardiac Troponin T. cMHC - Cardiac Heavy Chain Myosin. αSMA - α-sarcomeric-Actinin.


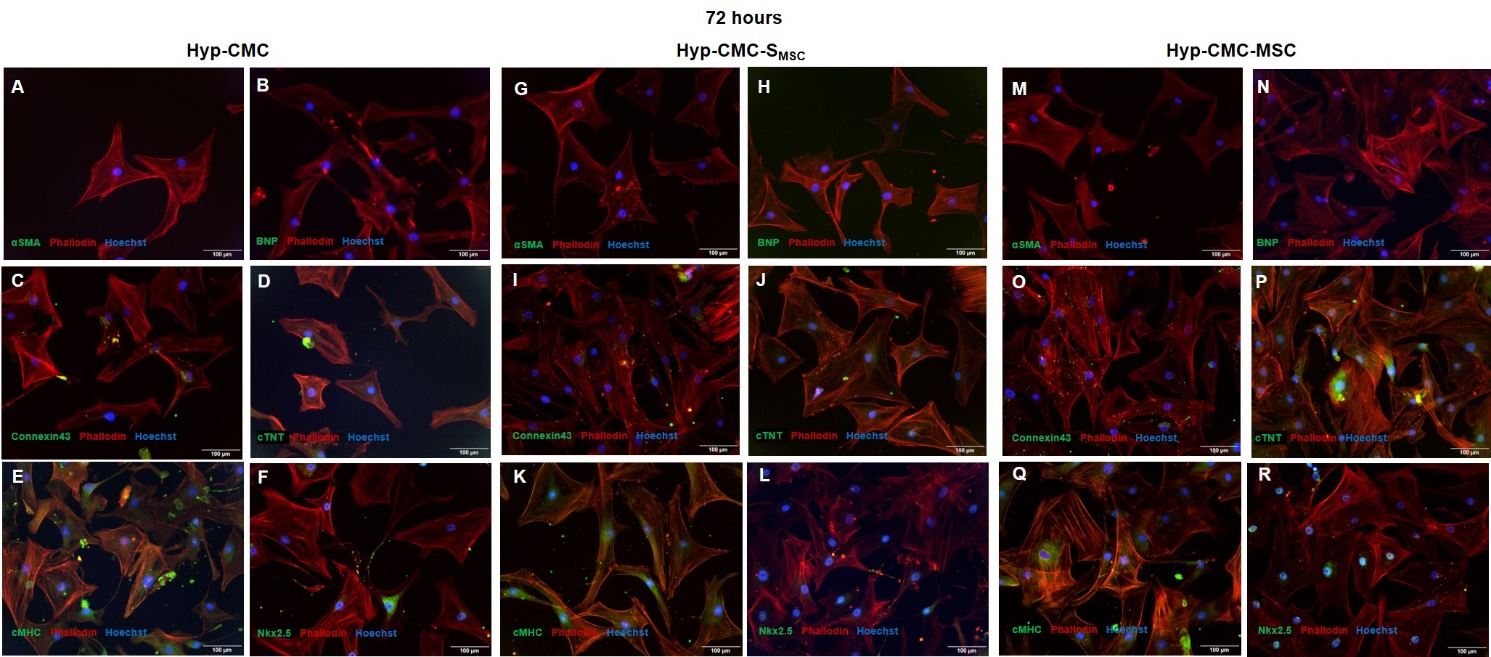


**Supplementary Figure 5:** **Characterization with immunofluorescence staining of CMC after 72 hours reoxygenation** from each of the three experimental groups: unstimulated hypoxic CMC (group Hyp-CMC, A-F), CMC stimulated with MSC secretome (group Hyp-CMC-S_MSC,_ G-L) and CMC stimulated directly with MSC (group Hyp-CMC-MSC, M-R). CMC showed no expression of αSMA (A, G, M) and BNP (B, H, N). Linage specific markers Connexin 43 (C, I, O), cTNT (D, J, P), cMHC (E, K, Q) and Nkx2.5 (F, L, R) were expressed throughout all setups. Cells were counterstained with Hoechst. MSC – Mesenchymal stem cells. CMC – cardiomyocytes. cTNT – Cardiac Troponin T. cMHC - Cardiac Heavy Chain Myosin. αSMA - α-sarcomeric-Actinin.
